# In a novel autoimmune and high-pressure glaucoma model a complex immune response is induced

**DOI:** 10.3389/fimmu.2024.1296178

**Published:** 2024-03-07

**Authors:** Sabrina Reinehr, Julien Wulf, Janine Theile, Kim K. Schulte, Marcus Peters, Rudolf Fuchshofer, H. Burkhard Dick, Stephanie C. Joachim

**Affiliations:** ^1^ Experimental Eye Research Institute, University Eye Hospital, Ruhr-University Bochum, Bochum, Germany; ^2^ Department of Molecular Immunology, Ruhr-University Bochum, Bochum, Germany; ^3^ Institute of Human Anatomy and Embryology, University Regensburg, Regensburg, Germany

**Keywords:** autoimmune glaucoma, complement system, microglia/macrophages, glaucoma animal models, immune response, intraocular pressure, retina, T-cells

## Abstract

**Background:**

The neurodegenerative processes leading to glaucoma are complex. In addition to elevated intraocular pressure (IOP), an involvement of immunological mechanisms is most likely. In the new multifactorial glaucoma model, a combination of high IOP and optic nerve antigen (ONA) immunization leads to an enhanced loss of retinal ganglion cells accompanied by a higher number of microglia/macrophages in the inner retina. Here, we aimed to evaluate the immune response in this new model, especially the complement activation and the number of T-cells, for the first time. Further, the microglia/macrophage response was examined in more detail.

**Methods:**

Six-week-old wildtype (WT+ONA) and βB1-connective tissue growth factor high-pressure mice (CTGF+ONA) were immunized with 1 mg ONA. A wildtype control (WT) and a CTGF group (CTGF) received NaCl instead. Six weeks after immunization, retinae from all four groups were processed for immunohistology, RT-qPCR, and flow cytometry, while serum was used for microarray analyses.

**Results:**

We noticed elevated numbers of C1q^+^ cells (classical complement pathway) in CTGF and CTGF+ONA retinae as well as an upregulation of *C1qa*, *C1qb*, and *C1qc* mRNA levels in these groups. While the complement C3 was only increased in CTGF and CTGF+ONA retinae, enhanced numbers of the terminal membrane attack complex were noted in all three glaucoma groups. Flow cytometry and RT-qPCR analyses revealed an enhancement of different microglia/macrophages markers, including CD11b, especially in CTGF and CTGF+ONA retinae. Interestingly, increased retinal mRNA as well as serum levels of the tumor necrosis factor α were found throughout the different glaucoma groups. Lastly, more T-cells could be observed in the ganglion cell layer of the new CTGF+ONA model.

**Conclusion:**

These results emphasize an involvement of the complement system, microglia/macrophages, and T-cells in glaucomatous disease. Moreover, in the new multifactorial glaucoma model, increased IOP in combination with autoimmune processes seem to enforce an additional T-cell response, leading to a more persistent pathology. Hence, this new model mimics the pathomechanisms occurring in human glaucoma more accurately and could therefore be a helpful tool to find new therapeutic approaches for patients in the future.

## 1 Introduction

An elevated intraocular pressure (IOP) is considered a main risk factor for glaucoma. However, it is incontrovertible that the immune system plays a major factor in its development (1, 2). Glaucoma itself is a multifactorial neurodegenerative disease leading to a loss of retinal ganglion cells (RGCs) and degeneration of the optic nerves (3). The aspect of its complexity makes it difficult to find appropriate therapies, besides lowering the IOP. Over the last years, it has been established that an activation of the immune system is involved in glaucomatous damage. For example, the initiation of the complement system was found to play a pivotal role in glaucomatous damage not only in different glaucoma animal models (4–9), but also in patients (10–12). It is known that the complement pathway and the resulting terminal complex serve as a cell surface or opsonization signal for the recognition by macrophages expressing the complement receptor C3r (13). In response to aging, retinal microglia/macrophages express complement proteins in retinae of rodents (14). After injuries, C1q, as part of the classical complement pathway, can also promote microglia activation, for example after ischemia/reperfusion or photo-oxidative damage (15, 16). Hence, the complement system and microglia/macrophages are in a lively crosstalk and might therefore reinforce neurodegenerative effects. Moreover, the complement system can modulate T-cell responses during the different immune response phases (17–19). In glaucoma, some studies point towards an involvement of T-cells. For example, transient elevation of the IOP resulted in a T-cell infiltration into the retina causing RGC loss (20). Further, an adoptive transfer of lymphocytes from glaucomatous mice into healthy ones stimulated RGC death (21).

All these studies indicate an interaction of complement, microglia/macrophages, and T-cells in glaucomatous damage. To confirm and assess these interactions, we evaluated the different cell types in a newly established multifactorial glaucoma animal model. We incorporated two risk factors to better mimic the complexity of the disease. In a first study, the high-pressure βB1-connective tissue growth factor (CTGF) mouse (22, 23) was combined with the normal-pressure experimental autoimmune glaucoma (EAG) model (24). Briefly, six-week-old wildtype (WT) and CTGF mice were immunized with an intraperitoneal injection of either the optic nerve antigen ONA (=WT+ONA, CTGF+ONA) or sodium chloride as control substance (=WT, CTGF). Previously, we were able to observe an additive optic nerve degeneration as well as a more advanced loss of RGCs in this new combination model, the CTGF+ONA mice. This was accompanied by more microglia/macrophage cells in the retina (25). Prior studies using the single models already revealed a contribution of the complement system. In CTGF mice, an activation of the complement system through C1q could be observed before RGC loss (9). In the EAG model, an increase in complement signaling, predominantly via the lectin pathway, was observed prior to RGC death (8, 26).

We now aimed to analyze the complement response and the T-cells in the new multifactorial model in comparison to the one-factor models at the time of RGC loss. Furthermore, we focused on different microglia/macrophage and complement system markers to further determine their role in glaucomatous damage. Hence, retinae of all groups were evaluated by flow cytometry, immunohistological stainings, and quantitative real-time PCR (RT-qPCR). Moreover, serum samples were used to perform microarray analysis. In terms of comparability, we used the same cDNA for RT-PCR as well as retinal cross-sections from the animals of the former publication (25).

We observed an activation of the complement system and an increase in different microglia/macrophage markers. Interestingly, this was not limited to the CTGF+ONA group, but was also noted in the single CTGF mice, while almost no alterations were observed in WT+ONA animals. These results suggest ongoing neuroinflammation solely in high-pressure glaucoma mice at this point in time. Intriguingly, an enhanced number of T-cells were only noted in the new CTGF+ONA mice. Hence, these findings underline the importance of using different glaucoma models to map a broad spectrum of this disease and thus can help to find new therapeutic approaches.

## 2 Methods

### 2.1 Animals

The ARVO statement for the use of animals in ophthalmic and vision research was followed for animals during all *in vivo* procedures. The animal care committee of North Rhine-Westphalia, Germany, approved all experiments concerning animals. Mice were kept under environmentally controlled conditions, including free access to water and food.

Transgenic CTGF and WT mice with a CD1 background were used in this study (9, 23, 27). For breeding, wildtype CD1 mice were obtained from Charles River (Sulzfeld, Germany) and CTGF mice were kindly provided by Prof. Dr. Fuchshofer (University Regensburg, Germany). The animal facility at the Ruhr-University Bochum (Bochum, Germany) was used for breeding and housing all the mice included in this study. Screening of potential CTGF mice was performed by genomic DNA isolation from tail biopsies and PCR testing of transgenic sequences using 5´-GGAAGTGCCAGCTCATCAGT-3´ and 5´-GTGCGGGACAGAAACCTG-3´ primers. This study included female and male mice.

### 2.2 Immunization

ONA was prepared and immunization was performed as previously described (28, 29). Intraperitoneal injections of ONA (1.0 mg/ml) were applied to six-week-old WT (=WT+ONA) and CTGF (=CTGF+ONA) mice by mixing the antigen with incomplete Freund’s adjuvant (50 µl; Sigma-Aldrich, St. Louis, MO, USA). NaCl injections with Freund’s adjuvant were applied to WT and CTGF (=CTGF) animals of the control groups. Moreover, on days 0 and 2, all mice were intraperitoneally injected with 1 µg pertussis toxin (Sigma-Aldrich) (25, 30).

### 2.3 Immunohistology and subsequent evaluations

Eyes were enucleated and fixed in 4% paraformaldehyde (PFA) for 1 h six weeks after immunization. Then, the tissues were treated with 30% sucrose and embedded in a Neg-50 compound (Tissue-Tek; Fisher Scientific, Schwerte, Germany). Cross-sections of the retina (10 µm) were cut with a cryostat (Fisher Scientific) for further staining (31).

Specific immunofluorescence antibodies were applied to identify different cell types (**Table 1**) (8). Briefly, retina cross-sections (n=7 retinae/group) were blocked with a solution containing 10-20% donkey, 2-3% bovine serum albumin, and/or goat serum, and 0.1% Triton-X in PBS. Sections were incubated with primary antibodies at room temperature overnight. Incubation using corresponding secondary antibodies was performed for 1 h on the next day. Nuclear staining with 4´,6 diamidino-2-phenylindole (DAPI, Serva Electrophoresis, Heidelberg, Germany) was included to facilitate the orientation on the slides. Negative controls were performed for each stain by using secondary antibodies only.

**Table 1:** Primary antibodies listed in alphabetical order and corresponding secondary antibodies used for immunohistology.

Primary antibodies					Corresponding secondary antibodies				
Antibody	Company	Order number	Concentration stock solution	Dilution	Antibody	Company	Dilution	Order number	Concentration stock solution
C1q	Abcam, Cambridge, UK	ab182451	Not stated	1:500	Donkey anti-rabbit Alexa Fluor 555	Invitrogen, Carlsbad, CA, USA	1:500	A31572	2.0 mg/ml
C3	Cedarlane, Burlington, ON, Canada	CL7334Ap	100 µg/ml	1:700	Goat anti-rabbit Cy3	Linaris, Dossenheim, Germany	1:500	ZRV1159	2.0 mg/ml
CD3-FITC	eBioscience, Waltham, MA, USA	14-0031-82	500 µg/ml	1:100	None (directly labelled)				
Iba1	Synaptic Systems, Göttingen, Germany	234009	1000 µg/ml	1:500	Donkey anti-chicken Alexa Fluor 488	Jackson Immuno Research, Ely, UK	1:500	703-545-155	1.5 mg/ml
					Donkey anti-chicken Cy3	Millipore, Burlington, MA, USA	1:500	AP194C	500 µg
MAC	Thermo Fisher, Waltham, MA, USA	BS-2673R	1000 µg/ml	1:200	Donkey anti-rabbit Alexa Fluor A555	Invitrogen, Carlsbad, CA, USA	1:500	A31572	2.0 mg/ml

The photographs were taken using a fluorescence microscope (Axio Imager M2, Zeiss, Oberkochen, Germany). Two photos of the peripheral and two of the central part of each retinal cross-section were captured (in total 24 images/animal). The images were transferred to Corel Paint Shop Pro (V13, Corel Corporation, Ottawa, Canada) and equal excerpts were cut out. Afterwards, C1q^+^, C3^+^, and membrane attack complex (MAC)^+^ cells were counted in the ganglion cell layer (GCL), inner plexiform layer (IPL), and inner nuclear layer (INL) using ImageJ software (NIH, Bethesda, MD, USA).

The number of CD3^+^ T-cells was assessed during microscopy. CD3^+^ cells were counted over the whole length of the retina for each cross-section. The number of T-cells was evaluated in the GCL, INL, and IPL.

### 2.4 Flow cytometry of retina samples

For flow cytometry, eyes were enucleated after six weeks. Both retinae of each animal were pooled in 1 ml of RPMI/10%FCS medium (n=4 samples/group) (32). The retinae were dispersed by vigorous pipetting. Afterwards, 6 ml medium was added, and tubes were centrifuged for 6 min (1200 rpm). The cell pellet was resuspended in a 3 ml enzyme solution (1 mg Collagenase I and 0.2 mg DNase in RPMI/10%FCS medium) and incubated for 60 min at 37°C. Subsequently, samples were dispersed, washed, filtered (40 µm), and suspended in 5 ml RPMI/10%FCS medium. Cells were counted using trypan blue (1:2 dilution) in an improved Neubauer counting chamber (VWR, Radnor, PA, USA). 1x10^5^ cells were placed in a 1.5 ml tube. Firstly, the Fcγ-receptors were blocked for 10 min (Thermo Fisher Scientific, Waltham, MA, USA). Afterwards, leukocytes were stained with CD45-FITC (0.25 µg), T-cells with CD4-PE (1.0 µg), and macrophages/microglia with CD11b-PE (1.0 µg). Corresponding isotype control staining was performed with rat IgG2b,κ-FITC and rat IgG2b,κ-PE (all: Thermo Fisher Scientific). The cells were washed and resuspended with 3% PFA. Afterwards, the autofluorescence signal was subtracted by gating on the isotype control and cells were counted with a Cyflow FACS (Partec, Görlitz, Germany). Further analysis was performed with Kaluza Analysis 2.1 (Beckman Coulter, Brea, CA, USA).

### 2.5 Quantitative real time PCR

At the end of the study, eyes were enucleated, and both retinae of each animal were pooled for RNA preparation and cDNA synthesis as previously described (n=4 samples/group) (23). The designed oligonucleotides for RT-qPCR are shown in **Table 2**. Expression was normalized against β-actin (*Actb*) and cyclophilin (*Ppid*) (29). The RT-qPCR was performed using DyNAmo Flash SYBR Green (Fisher Scientific) on the PikoReal RT-qPCR Cycler (Fisher Scientific) (33, 34). Values were transferred to REST^©^ software (Qiagen, Hilden, Germany) for further analysis.

**Table 2:** List of oligonucleotides used for mRNA expression analysis in retinae, while *Actb* and *Ppid* served as reference genes.

Gene	Forward (F) and reverse (R) oligonucleotides	GenBank acc. no.	Amplicon size
*Actb*-F *Actb*-R	ctaaggccaaccgtgaaag accagaggcatacagggaca	NM_007393.5	104 bp
*C1qa*-F *C1qa*-R	cgggtctcaaaggagagaga tcctttaaaacctcggatacca	NM_007572.2	71 bp
*C1qb*-F *C1qb*-R	aggcactccagggataaagg ggtcccctttctctccaaac	NM_009777.3	80 bp
*C1qc*-F *C1qc*-R	atggtcgttggacccagtt gagtggtagggccagaagaa	NM_007574.2	75 bp
*C3*-F *C3*-R	accttacctcggcaagtttct ttgtagagctgctggtcagg	NM_009778.3	75 bp
*Cd68*-F *Cd68*-R	tgatcttgctaggaccgctta taacggcctttttgtgagga	NM_001291058.1	66 bp
*Cfb*-F *Cfb*-R	ctcgaacctgcagatccac tcaaagtcctgcggtcgt	M57890.1	112 bp
*Hc*-F *Hc*-R	tgacaccaggcttcagaaagt agttgcgcacagtcagctt	XM_017315669.2	69 bp
*Itgam*-F *Itgam*-R	cctgtccctggctgtttcta accggagccatcaatcaaga	NM_001082960.1	208 bp
*Masp2*-F *Masp2*-R	ggcggctactattgctcct aacacctggcctgaacaaag	NM_001003893.2	86 bp
*Nos2-*F *Nos2-*R	ctttgccacggacgagac tcattgtactctgagggctgac	NM_010927.4	66 bp
*Ppid*-F *Ppid*-R	aaggatggcaaggattgaaa ctttaagcaattctgcctgga	NM_026352	95 bp
*Tgfb*-F *Tgfb*-R	aggaggtttataaaatcgacatgc tgtaacaactgggcagacagttt	XM_006497136.3	65 bp
*Tnf*-F *Tnf*-R	ctgtagcccacgtcgtagc ttgagatccatgccgttg	NM_013693.3	97 bp

The predicted amplicon sizes are given. F, forward; R, reverse; acc. no., accession number; bp, base pair.

### 2.6 Microarray of serum samples

Serum from each mouse was collected at the end of the study by heart punctation to investigate inflammatory cytokines. For each array, two blood samples were pooled to obtain the required volume (n=4 samples/group). Comprehensive analyses of inflammatory protein levels were performed by using the commercially available RayBio Mouse Inflammation Antibody Array 1 (RayBiotech, Norcross, GA, USA) as described previously (35–37). Briefly, for each sample, one nitrocellulose membrane, each containing 40 different antibodies in duplicate spots, were blocked, incubated with appropriately diluted sera (1:1), washed, and then incubated with a cocktail of biotin-conjugated antibodies specific for the different proteins. The chemiluminescent signal was detected using an imaging system (Fusion FX7 Edge; Vilber Lourmat, Eberhardzell, Germany). The resulting images were analyzed using the BIO-1D software (Vilber Lourmat) to measure the expression of various cytokines. Positive and negative control spots within the membranes were used to normalize the results from different membranes being compared.

### 2.7 Statistics

For immunofluorescence, data is displayed as mean ± standard error of the mean (SEM) and groups were compared by ANOVA followed by Tukey Honest post-hoc test (Statistica Software; Version 13, Dell, Tulsa, OK, USA). Regarding RT-qPCR, the relative expression values are presented as median ± quartile+minimum/maximum and were assessed via Pair Wise Fixed Reallocation Randomisation Test using REST^©^ software (Qiagen) (23, 24, 38). For flow cytometry, statistics comprised of Kruskal-Wallis test followed by Dunn’s test using Statistica and are presented as median ± interquartile range (IQR) ± range (25, 34). For microarray analysis, control values were set to 100% and data are presented as mean ± standard deviation (SD) ± SEM and WT+ONA, CTGF, and CTGF+ONA groups were each compared to the WT group by a non-parametric Mann-Whitney U test (Statistica) (34).

P-values below 0.05 were considered statistically significant, with *p<0.050, **p<0.010, and ***p<0.001 when compared to WT, ^#^p<0.050 and ^##^p<0.010 when compared to WT+ONA, and ^¥^p<0.05 when compared to CTGF.

## 3 Results

### 3.1 Elevated number of C1q^+^ cells in high-pressure animals

In the previous publication, elevated IOP was measured in CTGF and CTGF+ONA mice, while no changes were noted in WT+ONA animals. Furthermore, a loss of RGCs was reported in all three glaucoma groups, while it was even more pronounced in the novel CTGF+ONA mice (25). Now, we aimed to analyze the immune response in these animals more precisely. To investigate the classical pathway of the complement system in the retinae, immunohistological analyses of C1q were performed (39). Furthermore, co-staining with the microglia/macrophage marker Iba1 was utilized to elaborate a possible co-localization. Expression patterns of the subunits of C1q, namely *C1qa*, *C1qb*, and *C1qc* were evaluated via RT-qPCR. Each of these chains is required for a proper assembly of C1q (40). Moreover, the lectin pathway (mannose-binding serin protease 2) and the alternative pathway (complement factor B) were examined through RT-qPCR.

**Figure 1:**
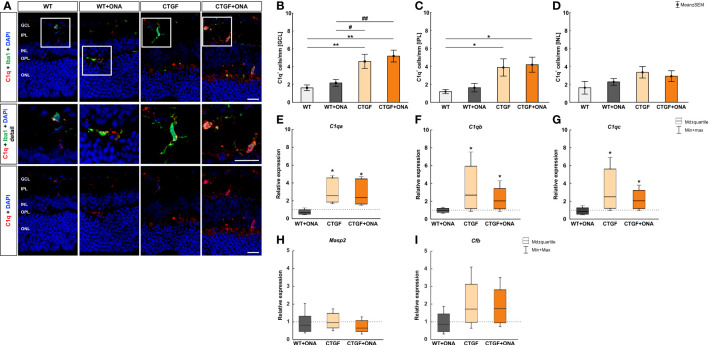
Elevated classical pathway in high-pressure animals. **(A)** Retinal cross-sections were labelled with an antibody against C1q (classical complement pathway; red) and cell nuclei were stained with DAPI (blue). An antibody against Iba1 (microglia/macrophages; green) was utilized to evaluate co-staining of C1q and Iba1. In the detailed images, it could be shown that many C1q^+^ cells are also positive for Iba1. **(B)** The number of C1q^+^ cells in the GCL was comparable in WT+ONA and WT animals. Contrary, significantly more C1q^+^ cells were noted in CTGF (p=0.007) and CTGF+ONA retinae (p=0.001) compared to WT ones. Also, this number was higher in CTGF (p=0.032) and CTGF+ONA mice (p=0.006) when compared to WT+ONA animals. **(C)** In the IPL, the number of C1q^+^ cells was similar in WT+ONA and WT mice. More C1q^+^ cells could be noted in CTGF(p=0.049) and CTGF+ONA retinae (p=0.025) compared to WT animals. **(D)** The number of C1q^+^ cells in the INL was comparable within all groups. **(E)** The mRNA expression of *C1qa* was not altered in WT+ONA mice. A significant upregulation of *C1qa* mRNA levels was observed in CTGF (p=0.039) and CTGF+ONA retinae (p=0.039). **(F)** The *C1qb* mRNA expression level did not differ in WT+ONA retinae compared to WT. In CTGF mice, a significant upregulated *C1qb* mRNA level was observed (p=0.035). Also, *C1qb* levels were elevated in CTGF+ONA mice (p=0.046). **(G)** While no alterations were revealed in the mRNA expression levels of *C1qc* in WT+ONA retinae, a significant upregulation was observable in CTGF (p=0.033) and CTGF+ONA mice (p=0.046). **(H)** Moreover, the *Masp2* mRNA levels, as part of the lectin pathway, were analyzed. Here, the *Masp2* expression was comparable in all glaucoma groups compared to WT retinae. **(I)** Also, the mRNA levels of the alternative pathway component *Cfb* were not altered within the groups. GCL, ganglion cell layer; IPL, inner plexiform layer; INL, inner nuclear layer; OPL, outer plexiform layer; ONL, outer nuclear layer. Values in **(B–D)** are mean ± SEM and in **(E–I)** median ± quartile ± minimum/maximum. The dotted lines in **(E–I)** represent the relative expression of the WT group. Scale bars: 20 µm; scale bar in detailed images: 10 µm. For immunohistology: n=7 retinae/group, for RT-qPCR: n=4 samples/group. *p<0.050 and **p<0.010 *vs.* WT; ^#^p<0.050 and ^##^p<0.010 *vs*. WT+ONA.

The number of C1q^+^ cells was counted in the GCL, IPL, as well as INL in all groups. Co-staining with Iba revealed many C1q^+^ microglia/macrophages (**Figure 1A**). The number of C1q^+^ cells in the GCL was comparable in WT+ONA (2.18 ± 0.39 cells/mm) and WT animals (1.63 ± 0.32 cells/mm; p=0.902). However, significantly more C1q^+^ cells were noted in CTGF (4.59 ± 0.79 cells/mm; p=0.007) and CTGF+ONA retinae (5.20 ± 0.67 cells/mm; p=0.001) compared to WT ones. Also, this number was higher in CTGF (p=0.032) and CTGF+ONA mice (p=0.006) when compared to WT+ONA animals (**Figure 1B**). In the IPL, the number of C1q^+^ cells did not differ between WT+ONA (1.63 ± 0.47 cells/mm) and WT animals (1.19 ± 0.23 cells/mm; p=0.969). Similar to the GCL, a significantly increased number of C1q^+^ cells was seen in CTGF (3.88 ± 0.96 cells/mm; p=0.049) as well as CTGF+ONA retinae (4.19 ± 0.83; p=0.025) compared to WT ones. A trend towards more C1q^+^ cells was noted in CTGF+ONA mice compared to WT+ONA animals (p=0.065), but not compared to CTGF ones (p=0.989; **Figure 1C**). The cell counts of C1q in the INL revealed no alterations within the groups. The cell number was comparable in WT+ONA (2.27 ± 0.37 cells/mm; p=0.864), CTGF (3.33 ± 0.64 cells/mm; p=0.198), as well as CTGF+ONA mice (2.91 ± 0.60 cells/mm; p=0.430) when compared to WT ones (1.62 ± 0.71 cells/mm). Consequently, no difference was noted in the number of C1q^+^ cells when comparing WT+ONA (p=0.869) as well as CTGF mice (p=0.956) to CTGF+ONA animals (**Figure 1D**).

Further, the mRNA levels of the C1q subunits, namely *C1qa*, *C1qb*, and *C1qb*, were analyzed. Corresponding to the immunohistological results, the mRNA expression of *C1qa* was not altered in WT+ONA mice (0.71-fold expression; p=0.118). A significant upregulation of *C1qa* mRNA levels was observed in CTGF (2.59-fold expression; p=0.039) and CTGF+ONA retinae (2.36-fold expression; p=0.039; **Figure 1E**). Further, the mRNA expression level of *C1qb* did not differ in WT+ONA compared to WT retinae (0.99-fold expression; p=0.890). In CTGF mice, a significantly upregulated mRNA level of *C1qb* was observed (2.69-fold expression; p=0.035). Also, *C1qb* levels were elevated in CTGF+ONA mice (2.04-fold expression; p=0.046; **Figure 1F**). Similar results were obtained for the mRNA expression levels of *C1qc*. While no alterations were revealed in WT+ONA retinae (0.83-fold expression; p=0.391), a significant upregulation was observable in CTGF (2.5-fold expression; p=0.033) as well as in CTGF+ONA mice (2.06-fold expression; p=0.046; **Figure 1G**).

Next, the lectin as well as the alternative pathway of the complement system were investigated using RT-qPCR. Here, the mRNA expression levels of *Masp2* were neither altered in WT+ONA retinae (0.82-fold expression; p=0.592) nor in CTGF (0.95-fold expression; p=0.916) or in CTGF+ONA mice (0.652-fold expression; p=0.306) compared to WT ones (**Figure 1H**). Also, no changes in the mRNA expression levels of *Cfb* could be noted in WT+ONA (0.85-fold expression; p=0.588), CTGF (1.72-fold expression; p=0.262), and CTGF+ONA mice (1.74-fold expression; p=0.212) compared to WT retinae (**Figure 1I**).

### 3.2 More C3 in CTGF and CTGF+ONA mice

To evaluate if the complement system is activated in general, the factor C3, as part of the terminal complement pathway (41), was examined in all four groups immunohistologically as well as through RT-qPCR analyses. As addition, co-staining with the microglia/macrophage marker Iba1 was utilized to elaborate a possible co-localization.

**Figure 2:**
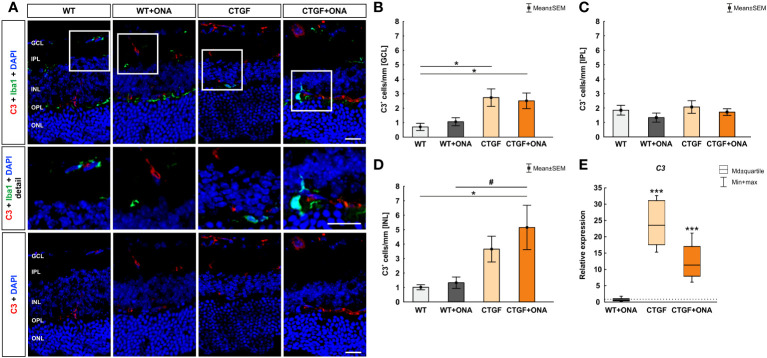
Increase of terminal complement pathway components. **(A)** An anti-C3 antibody (terminal complement pathway) was used to label retinal cross-sections (red), while DAPI counterstained cell nuclei (blue). An antibody against Iba1 (microglia/macrophages; green) was utilized to show co-staining of C3 and Iba1. Herein, most C3 cells were not positive for Iba1^+^ microglia (detailed images). **(B)** The number of C3^+^ cells in the GCL was comparable in WT+ONA and WT mice. Significantly more C3^+^ cells were noted in CTGF (p=0.018) and CTGF+ONA mice (p=0.039) compared to WT ones. **(C)** The cell counts of C3 in the IPL were comparable in all groups. **(D)** In the INL, the number of C3^+^ cells did not differ between WT+ONA and CTGF mice compared to WT ones. A higher number of C3^+^ cells was noted in CTGF+ONA retinae compared to WT (p=0.019) and WT+ONA mice (p=0.032). **(E)** No alterations were measured in *C3* mRNA expression levels in WT+ONA animals. In CTGF as well as in CTGF+ONA mice, a significant upregulation of *C3* mRNA levels could be observed (both: p<0.001). GCL, ganglion cell layer; IPL, inner plexiform layer; INL, inner nuclear layer; OPL, outer plexiform layer; ONL, outer nuclear layer. Values in **(B–D)** are mean ± SEM and in **(E)** median ± quartile ± minimum/maximum. The dotted line in **(E)** represents the relative expression of the WT group. Scale bars: 20 µm, scale bar in detailed images: 10 µm. For immunohistology: n=7 retinae/group, for RT-qPCR: n=4 samples/group. *p<0.050 and ***p<0.001 *vs.* WT; ^#^p<0.050 *vs*. WT+ONA.

In the immunohistology, the number of C3^+^ cells were counted in the GCL, IPL, and INL. The co-staining of Iba and C3 showed only few co-localized cells (**Figure 2A**). In the GCL, the number of C3^+^ cells were comparable in WT+ONA (1.06 ± 0.28 cells/mm) and WT mice (0.70 ± 0.25 cells/mm; p=0.939). Significantly more C3^+^ cells were noted in CTGF (2.73 ± 0.60 cells/mm; p=0.018) and CTGF+ONA mice (2.51 ± 0.54 cells/mm; p=0.039) compared to WT ones. No differences were detected when comparing WT+ONA (p=0.128) and CTGF retinae (p=0.985) to the CTGF+ONA group (**Figure 2B**). In the IPL, C3^+^ cells did not differ in WT+ONA (1.34 ± 0.31 cells/mm; p=0.709), CTGF (2.07 ± 0.43 cells/mm; p=0.997), and CTGF+ONA retinae (1.72 ± 0.23 cells/mm; p=0.992) compared to WT mice (1.85 ± 0.34 cells/mm). No differences were detected when comparing WT+ONA (p=0.856) and CTGF retinae (p=0.880) to the CTGF+ONA group (**Figure 2C**). The evaluation of C3^+^ cells in the IPL revealed no alterations in the WT+ONA (1.33 ± 0.39 cells/mm; p=0.995) and CTGF group (3.65 ± 0.89 cells/mm; p=0.199) when compared to WT mice (1.01 ± 0.17 cells/mm). Significantly more C3^+^ cells were observed in CTGF+ONA retinae (5.15 ± 1.53 cells/mm) compared to WT (p=0.019) as well as to WT+ONA animals (p=0.032), but not the CTGF mice (p=0.657; **Figure 2D**).

The mRNA levels of *C3* were not altered in WT+ONA animals (0.62-fold expression; p=0.256). In CTGF (23.53-fold expression; p<0.001) as well as in CTGF+ONA mice (11.34-fold expression; p<0.001) a significant upregulation of *C3* mRNA levels could be observed (**Figure 2E**).

### 3.3 Enhanced number of MAC^+^ deposits in all glaucoma animals

Conclusively, we examined the expression of the terminal complement complex MAC (41) in the retinae via immunohistological stainings. In addition, the mRNA levels of the hemolytic complement (*Hc*), as a part of MAC, were evaluated through RT-qPCR. Moreover, co-staining with the microglia/macrophage marker Iba1 was used to show a possible co-localization with MAC^+^ cells.

**Figure 3:**
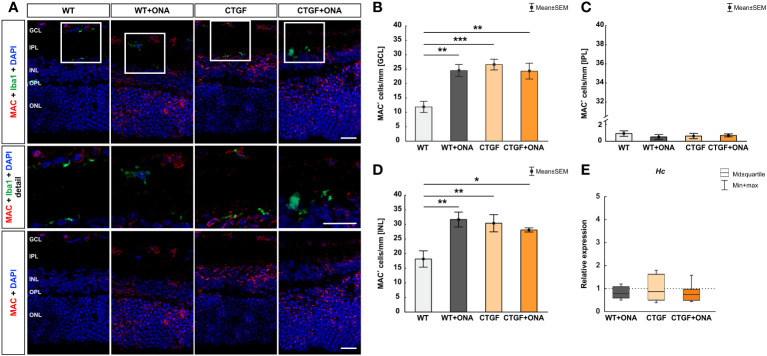
More MAC^+^ cells in all glaucoma groups. **(A)** Retinae were stained with an antibody against MAC (terminal complement complex; red) and cell nuclei were labelled with DAPI (blue). An antibody against Iba1 (microglia/macrophages; green) was used to show co-staining of MAC and Iba1. Mostly, MAC^+^ cells were not co-stained with microglia/macrophages (detailed images). **(B)** Regarding cell counts in the GCL, significantly more MAC^+^ deposits were noted in WT+ONA (p=0.002), CTGF (p<0.001), and CTGF+ONA mice (p=0.003) compared to WT ones. **(C)** In the IPL, the number of MAC^+^ cells was similar within all groups. **(D)** In the INL, significantly more MAC^+^ cells were observed in WT+ONA (p=0.003), CTGF (p=0.008), and CTGF+ONA mice (p=0.037) compared to WT animals. **(E)** The mRNA expression levels of *Hc* were not altered within all groups. GCL, ganglion cell layer; IPL, inner plexiform layer; INL, inner nuclear layer; OPL, outer plexiform layer; ONL, outer nuclear layer. Values in **(B–D)** are mean ± SEM and in **(E)** median ± quartile ± minimum/maximum. The dotted line in **(E)** represents the relative expression of the WT group. Scale bars: 20 µm, scale bar in detailed images: 10 µm. For immunohistology: n=7 retinae/group, for RT-qPCR: n=4 samples/group. *p<0.050; **p<0.010, and ***p<0.001 *vs.* WT.

The number of MAC^+^ cells was counted in the GCL, IPL, and INL of all groups. Barely any MAC^+^ cells were co-localized with Iba1^+^ microglia/macrophages (**Figure 3A**). In the GCL of WT+ONA mice, significantly more MAC^+^ cells could be detected (24.52 ± 2.09 cells/mm) compared to WT animals (11.87 ± 1.94 cells/mm; p=0.002). Further, more MAC^+^ deposits were noted in CTGF mice (26.60 ± 1.86 cells/mm) in comparison to WT ones (p<0.001). Similarly, the number of MAC^+^ cells was increased in CTGF+ONA retinae (24.33 ± 2.76 cells/mm; p=0.003). No changes were noted when comparing WT+ONA (p=1.000) or CTGF mice (p=0.883) to the CTGF+ONA group (**Figure 3B**). In the IPL, the number of MAC^+^ cells did not differ in WT+ONA (0.48 ± 0.26 cells/mm; p=0.750), CTGF (0.57 ± 0.28 cells/mm; p=0.880), and CTGF+ONA group (0.64 ± 0.16 cells/mm; p=0.946) compared to WT mice (0.84 ± 0.30 cells/mm). Also, no alterations were observed by comparing WT+ONA (p=0.969) and CTGF animals (p=0.997) to CTGF+ONA retinae (**Figure 3C**). The MAC cell counts in the INL revealed significantly more cells in WT+ONA (31.67 ± 2.56 cells/mm; p=0.003), CTGF (30.39 ± 2.94 cells/mm; p=0.008) and CTGF+ONA mice (28.10 ± 0.71 cells/mm; p=0.037) when compared to WT ones (18.16 ± 2.80 cells/mm). The number of MAC^+^ cells was not different when comparing WT+ONA (p=0.727) and CTGF animals (p=0.907) to CTGF+ONA retinae (**Figure 3D**).

The *Hc* mRNA expression levels were not altered in WT+ONA (0.79-fold expression; p=0.249), CTGF (0.87-fold expression; p=0.604), or CTGF+ONA retinae (0.75-fold expression; p=0.181; **Figure 3E**).

### 3.4 Increased microglia and macrophage response in CTGF and CTGF+ONA retinae

In the previous publication, increased numbers of Iba1^+^ microglia/macrophages were noted in CTGF and CTGF+ONA retinae, while more Tmem119^+^ and Iba1^+^ microglia were only observed in CTGF+ONA mice (25). Hence, in the present study, the responses of microglia and macrophages should be examined more precisely. Therefore, flow cytometry was used to detect CD45^+^ and CD11b^+^ cells in all groups.

**Figure 4:**
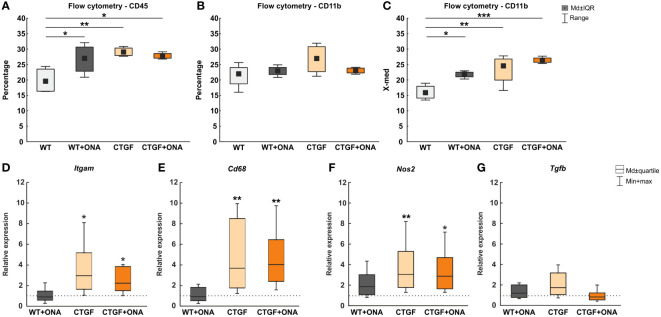
Enhanced microglia and macrophage markers in CTGF and CTGF+ONA animals. **(A)** Significantly more CD45^+^ cells were observed in WT+ONA (p=0.036), CTGF (p=0.006), and CTGF+ONA mice (p=0.016) compared to WT. **(B)** Further, the percentage of CD11b^+^ microglia cells was determined using flow cytometry. Here, no changes could be observed within all groups. **(C)** In addition, the fluorescent intensity of CD11b was measured. Here, a significantly higher intensity was detected in WT+ONA (p=0.036), CTGF (p=0.008) and CTGF+ONA retinae (p<0.001) when compared to WT ones. **(D)** The *Itgam* (CD11b) mRNA expression levels were unchanged in WT+ONA retinae. However, a significant upregulation was measured in CTGF (p=0.027) as well as in CTGF+ONA samples (p=0.042). **(E)** While no alterations could be revealed regarding *Cd68* mRNA expression levels in WT+ONA mice, significantly upregulated expression levels were noted in both CTGF and CTGF+ONA retinae (both: p=0.009). **(F)** No changes were observed in *Nos2* mRNA expression levels in WT+ONA mice. The *Nos2* mRNA expression levels were significantly upregulated in CTGF (p=0.008) and CTGF+ONA animals (p=0.023). **(G)** The mRNA expression of *Tgfb* was comparable to the WT situation in all groups. Values in **(A–C)** are median ± interquartile range ± range and in **(D–G)** median ± quartile ± minimum/maximum. The dotted lines in **(D–G)** represent the relative expression of the WT group. n=4 samples/group. *p<0.050, **p<0.010, and ***p<0.001 *vs.* WT.

In WT+ONA mice with a median number of 26.98 (IQR 22.81-30.65) a significantly higher percentage of CD45^+^ cells was noted when compared to WT ones (19.52, IQR 16.35-23.49; p=0.036). More CD45^+^ cells were observed in CTGF (29.01, IQR 27.93-30.34; p=0.006) and CTGF+ONA mice (27.66, IQR 27.05-28.55; p=0.016) compared to WT animals (**Figure 4A**).

Further, the percentage of CD11b^+^ cells was counted using FACS. Here, no changes could be observed in WT+ONA (22.93, IQR 21.73-24.06; p=0.855), CTGF (26.93, IQR 22.70-30.78; p=0.099), and CTGF+ONA retinae (23.11, IQR 22.27-23.82; p=0.819) compared to WT ones (21.94, IQR 18.74-24.02; **Figure 4B**).

In addition to the percentage of CD11b^+^ labelled cells, the fluorescent intensity of CD11b was measured. Here, a significantly higher CD11b fluorescent intensity was noted in WT+ONA retina (21.88, IQR 14.12-17.95) compared to WT animals (15.86, IQR 14.12-17.95; p=0.036). Also, a significantly higher intensity was seen in CTGF (24.54, IQR 19.95-26.78; p=0.008) and CTGF+ONA retinae (26.29, IQR 25.71-27.11; p<0.001) when compared to WT ones (**Figure 4C**).

Moreover, RT-qPCR was used to examine the mRNA levels of different microglia and macrophage markers. Here, the *Itgam* (CD11b) mRNA levels were not altered in WT+ONA retinae (0.91-fold expression; p=0.810). Contrary, a significant upregulation of *Itgam* was observable in CTGF (2.97-fold expression; p=0.027) as well as in CTGF+ONA mice (2.24-fold expression; p=0.042; **Figure 4D**).

The mRNA expression levels of *Cd68* (macrophages/resident microglia) in WT+ONA animals were comparable to the WT situation (0.91-fold expression; p=0.835). In CTGF retinae, a significant upregulation of *Cd68* mRNA levels could be revealed (3.67-fold expression, p=0.009). Also, significantly elevated *Cd68* mRNA levels were notable in CTGF+ONA samples (4.03-fold expression; p=0.009; **Figure 4E**).

No changes were seen in the mRNA expression levels of *Nos2* (iNOS) in WT+ONA mice (1.85-fold expression; p=0.140). The *Nos2* mRNA expression levels were significantly upregulated in CTGF (3.05-fold expression; p=0.008) and CTGF+ONA animals (2.87-fold expression; p=0.023; **Figure 4F**).

The mRNA expression of *Tgfb* (transforming growth factor-β2 (TGF-β2)) was comparable to the WT situation in WT+ONA mice (1.20-fold expression; p=0.376). A trend towards an upregulation was detected in CTGF animals (1.73-fold expression; p=0.055). No changes in the *Tgfb* mRNA levels were seen in CTGF+ONA mice (0.82-fold expression; p=0.484; **Figure 4G**).

### 3.5 Elevated levels of tumor necrosis factor-α

In serum samples of glaucoma patients, increased levels of tumor necrosis factor-α (TNF-α) could be detected (42, 43). Hence, we checked the retinas as well as the serum samples of our animals regarding this factor and its receptors.

**Figure 5:**
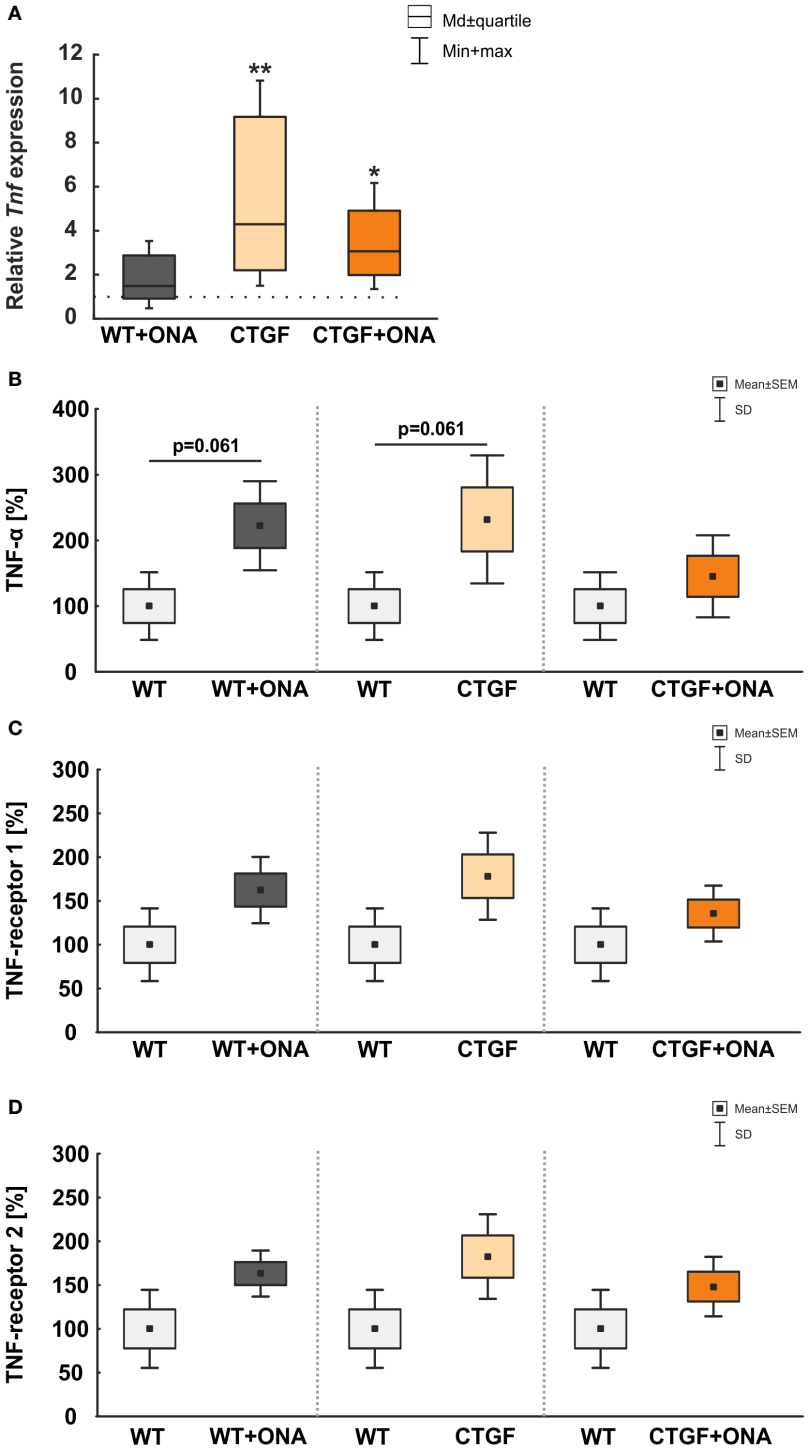
Elevated TNF-α levels. **(A)** The mRNA expression level of *Tnf* was not altered in WT+ONA mice. A significant *Tnf* upregulation was noted in CTGF (p=0.006) and CTGF+ONA retinae (p=0.026). **(B)** The microarray serum analyses showed no significant changes for TNFα in all groups. **(C)** The serum levels of TNF-receptor 1 were not significantly altered in WT+ONA and CTGF mice in comparison to WT samples. Also, the CTGF+ONA mice displayed no alterations in the serum TNF-receptor 1 levels. **(D)** The serum levels of TNF-receptor 2 were not altered in all glaucoma groups compared to WT controls. Values in **(A)** are median ± quartile ± minimum/maximum and in **(B–D)** mean ± SD ± SEM. The dotted line in **(A)** represents the relative expression of the WT group. n=4 samples/group. *p<0.050 and **p<0.010 *vs.* WT.

In the retina, the mRNA expression level of *Tnf* was not altered in WT+ONA mice (1.49-fold expression; p=0.302). A significant upregulation of *Tnf* was noted in CTGF (4.30-fold expression; p=0.006) and CTGF+ONA retinae (3.07-fold expression; p=0.026; **Figure 5A**).

Furthermore, microarray analyses of serum samples were performed. Here, the examination showed a trend towards higher levels of TNF-α in WT+ONA serum (222.38 ± 33.90%) compared to the WT group (100.00 ± 25.74%; p=0.061). Moreover, a trend towards higher TNF-α levels were observed in CTGF animals (232.03 ± 48.75%) in comparison to WT mice (p=0.061), whereas no changes were detected in CTGF+ONA serum (145.39 ± 31.22%) when compared to WT animals (p=0.470; **Figure 5B**).

Neither WT+ONA (162.43 ± 18.94%; p=0.112), CTGF (178.28 ± 24.86%; p=0.112), nor CTGF+ONA mice (135.62 ± 15.91%; p=0.312) displayed alterations in the serum TNF-receptor 1 levels when compared to WT samples (100.00 ± 20.74%; **Figure 5C**).

The serum levels of TNF-receptor 2 were not altered in WT+ONA (163.15 ± 13.15%; p=0.112), CTGF (182.57 ± 24.14%; p=0.112), and CTGF+ONA mice (148.27 ± 17.02%; p=0.112) when compared to WT animals (100.00 ± 22.26%; **Figure 5D**).

### 3.6 Enhanced macrophage associated proteins in the serum

To deepen the understanding of cytokine levels in the sera of the different animal models, microarray analyses were performed.

**Figure 6:**
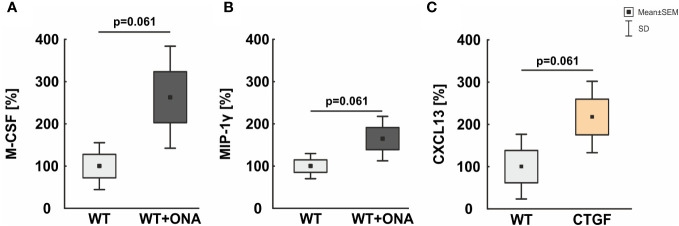
Mild increase of macrophage associated proteins in serum. **(A)** In the serum of WT+ONA animal no significant changes in the levels M-CSF was noted. **(B)** No significant alterations were observed in serum levels of MIP-1γ in WT+ONA animals compared to WT mice. **(C)** The serum levels of CXCL13 were not altered significantly in CTGF samples compared to WT mice. Values are mean ± SD ± SEM. N=4 samples/group.

We could show that a trend towards an upregulation of the macrophage colony-stimulating factor (M-CSF) could be noted in the serum of WT+ONA mice (263.10 ± 60.38%) compared to WT ones (100.00 ± 27.76%; p=0.061; **Figure 6A**).

Further, a trend towards higher serum levels of the macrophage inflammatory protein-1 gamma (MIP-1γ) was noted in WT+ONA animals (165.10 ± 26.28%) compared to WT mice (100.00 ± 14.87%; p=0.061; **Figure 6B**).

The serum levels of the C-X-C motif chemokine 13 (CXCL13) were twice as high in CTGF serum (217.54 ± 42.26%) then in WT mice (100.00 ± 38.32%), but only a trend towards a significant difference was detected (p=0.061; **Figure 6C**).

### 3.7 More T-cells in the GCL of CTGF+ONA mice

Previous reports implicate a T-cell response in glaucoma pathogenesis (20, 44, 45). Hence, we aimed to determine whether the pan T-cell marker CD3 could be detected in the retinae of the experimental groups via immunohistology. In addition, co-staining with the microglia/macrophage marker Iba1 was performed to elaborate a possible co-localization. Further, flow cytometry was utilized to examine the number of CD4^+^ T helper cells.

**Figure 7:**
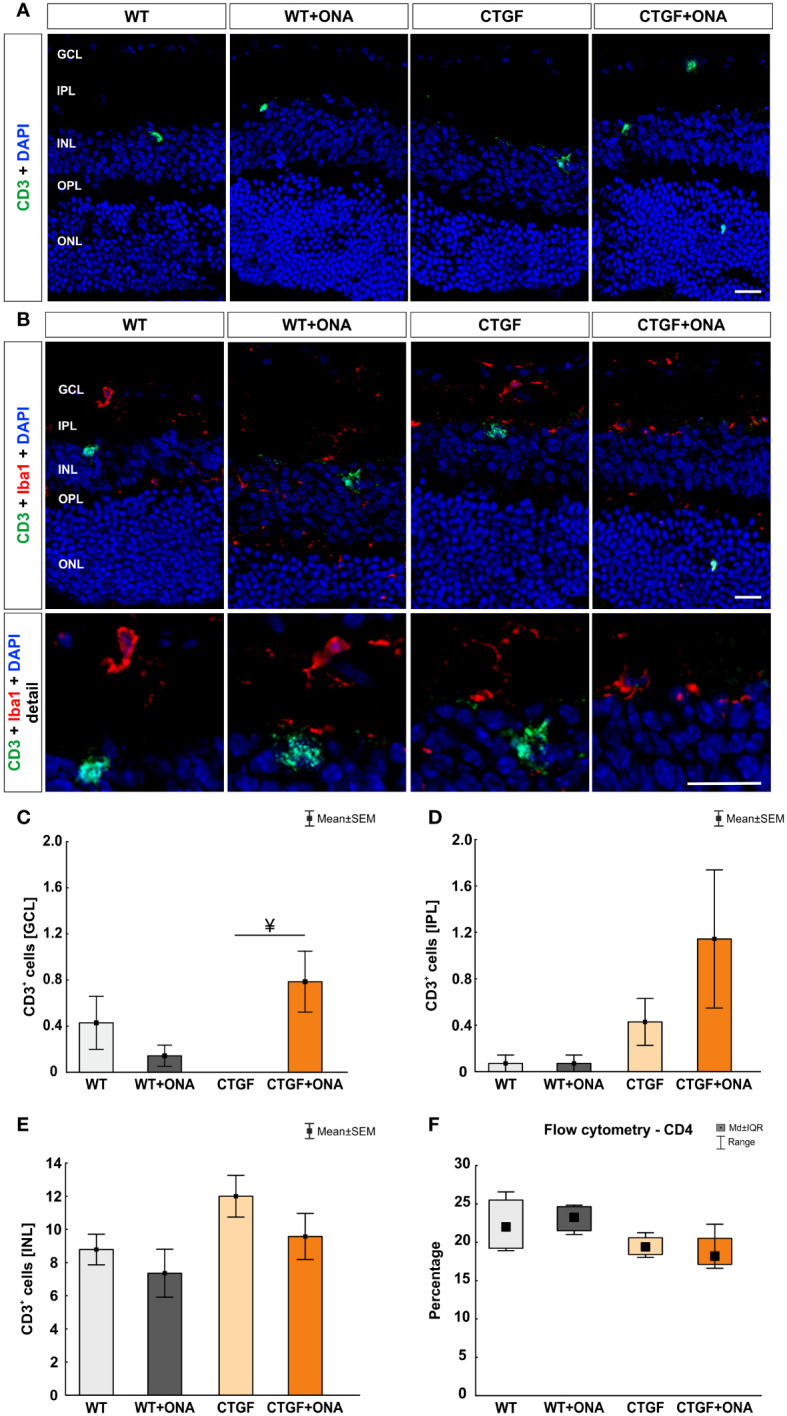
More T-cells in the GCL of CTGF+ONA mice. **(A)** Retinae of all groups were labelled with an antibody against CD3 to detect pan T-cells (green). DAPI counterstained cell nuclei (blue). **(B)** Additionally, an antibody against Iba1 (microglia/macrophages; red) was used to investigate possible co-staining of CD3 (green) and Iba1. Cell nuclei were labelled with DAPI (blue). The staining revealed that T-cells were predominantly not co-labelled with Iba^+^ microglia/macrophages. **(C)** In the GCL, the number of T-cells remained unchanged in WT+ONA, CTGF, and CTG+ONA mice compared to WT ones. Significantly more CD3^+^ cells were noted in CTGF+ONA mice compared to the CTGF group (p=0.025). **(D)** The number of CD3^+^ T-cells was not altered within the groups counted in the IPL. **(E)** Also, no changes could be detected in the INL regarding the number of T-cells. **(F)** Flow cytometry of CD4^+^ T-helper cells revealed no alterations within all groups. GCL, ganglion cell layer; IPL, inner plexiform layer; INL, inner nuclear layer; OPL, outer plexiform layer; ONL, outer nuclear layer. Values in **(C–E)** are mean ± SEM and in **(F)** mean ± interquartile range ± range. Scale bars: 20 µm, scale bar in detailed images: 10 µm. For immunohistology: n=7 retinae/group, for flow cytometry: n=4 samples/group. ^¥^p<0.050 *vs.* CTGF.

The number of CD3^+^ T-cells was counted in the GCL, IPL, and INL of each group. The co-staining of Iba1 and CD3 showed no co-localized cells (**Figures 7A, B**). In the GCL, no changes in the number of T-cells were noted in WT+ONA (0.14 ± 0.09 cells/GCL; p=0.683), CTGF (0.00 ± 0.00 cells/GCL; p=0.358), and CTGF+ONA mice (0.78 ± 0.26 cells/GCL; p=0.514) compared to WT animals (0.43 ± 0.23 cells/GCL). About 82% more cells were seen in CTGF+ONA retinae compared to WT+ONA ones, but this difference was not significant (p=0.083). Significantly more CD3^+^ cells were noted in CTGF+ONA mice compared to the CTGF group (p=0.025; **Figure 7C**). Concerning CD3^+^ cells in the IPL, no alterations could be noticed in WT+ONA (0.07 ± 0.07 cells/IPL; p=1.000), CTGF (0.43 ± 0.20 cells/IPL; p=0.957), as well as CTGF+ONA retinae (1.14 ± 0.59 cells/IPL; p=0.108) compared to WT animals (0.07 ± 0.07 cells/IPL). Also, the number of CD3^+^ cells was similar in WT+ONA (p=0.108) and CTGF mice (p=0.404) in comparison to CTGF+ONA retinae (**Figure 7D**). In the INL, a higher number of CD3^+^ T-cells could be seen. However, there was no difference within the groups. In WT+ONA (7.36 ± 1.45 cells/INL; p=0.856), CTGF (12.00 ± 1.26 cells/INL; p=0.303), and CTGF+ONA mice (9.57 ± 1.39 cells/INL; p=0.972), the number of CD3^+^ cells was comparable to the WT group (8.790 ± 0.92 cells/INL). Further, no difference was noted when comparing WT+ONA (p=0.614) and CTGF mice (p=0.541) to the CTGF+ONA retinae (**Figure 7E**).

CD4^+^ T-cells were counted using flow cytometry. Here, the median percentage of cells was comparable in WT+ONA (23.24, IQR 21.53-24.64; p=0.958), CTGF (19.37, IQR 18.42-20.59; p=0.299), and CTGF+ONA animals (18.16, IQR 17.12-20.53; p=0.166) when compared to WT retinae (21.99, IQR 19.23-25.51; **Figure 7F**).

## 4 Discussion

The aging of the society will consequently lead to more cases of age-dependent neurodegenerative diseases, including glaucoma. This, in turn, increases the demand of effective diagnostic and therapeutic options. Therefore, further comprehension of the fundamental pathomechanisms resulting in glaucoma is necessary, which can be achieved by using suitable animal models. Since most glaucoma models mimic mainly one pathogenic factor, such as elevated IOP, excitotoxity, or immune response, we implemented a new multifactorial model. Here, the combination of the high-pressure CTGF mouse and the normal-pressure EAG model led to an enhanced RGC loss and optic nerve degeneration. Furthermore, elevated numbers of microglia/macrophages were observed in the retinae of this novel model (25). We now aimed to characterize the immune response in these animals more precisely, especially microglia/macrophages and complement pathway. Additionally, we analyzed T-cells in the novel model for the first time.

In the central nervous system, microglia/macrophages are key mediators of occurring inflammation (46). When becoming reactive, they are able to secrete pro-inflammatory cytokines and complement factors, including TNF and C1q (47). C1q can initiate the classical pathway of the complement system and ultimately provoke the formation of the terminal complex MAC. During the development of the brain, C1q drives synaptic pruning to shape neuronal circuitry by binding to synapses and tagging them for phagocytosis by microglia cells (48, 49). It is postulated that similar effects occur in neurodegenerative diseases. Here, aging or other insults seem to re-activate this mechanism leading to a loss of synapses and consequently to neuronal cell death. In human glaucoma, increased levels of C1q could be detected in the retina as well as in the vitreous (10, 50, 51). In addition, more C1q was observed in different glaucoma animal models (4, 9, 50). Hereby, it appears that C1q is mostly activated in ocular hypertension (OHT) models. This is in accordance with the study presented here. Enhanced numbers of C1q^+^ cells as well as increased mRNA levels were only detected in CTGF and CTGF+ONA mice, both groups have elevated IOP, while no alterations were observed in WT+ONA retinae. Previous studies in the normal-pressure EAG model revealed that the activation of the complement system is mostly driven by the lectin pathway in these animals (8, 26).

While more C3^+^ cells were also solely noted in CTGF and CTGF+ONA retinae, a higher number of MAC^+^ cells was observed in all three glaucoma groups. Generally, nucleated cells are protected from lysis by MAC. However, a high-density of MAC pores can lead to the injury or death of these cells, whereas low-density assembly of MAC on some cell surfaces results in a pro-inflammatory response (52–55). There is clear evidence of microglia expressing numerous complement genes (56, 57). In our previous study, where we implemented the new multifactorial glaucoma model, increased numbers of Iba1^+^ microglia/macrophages were noted in CTGF and CTGF+ONA retinae, while more Tmem119^+^ and Iba1^+^ microglia could be observed in CTGF+ONA mice (25). Now, co-staining of complement markers with Iba1 showed that C1q was often co-localized with Iba1^+^ microglia/macrophages, while only few C3^+^ or MAC^+^ cells showed co-localization with Iba1. Similar results were noted in a porcine organ culture model, where damage was induced by oxidative stress or hypoxia (58). It is well known that microglia can express complement proteins, including C1q (56, 59, 60). Assumingly, microglia were attracted by chemotactic signals and subsequently expressed C1q. Its binding to an activator initiates a cascade of enzymatic reactions resulting in the opsonization of the activating substance, the generation of the activation peptides C3a and C5a, and the formation of a membranolytic pore. Moreover, binding of C3a and C5a to their cellular receptors contributes to inflammation (59, 61).

As stated before, microglia/macrophages play a pivotal role in neurodegenerative diseases. Hence, we aimed to investigate these cells in more detail. By using flow cytometry, we detected higher cell counts of CD45^+^ as well as an elevated intensity of CD11b^+^ cells in retinae of CTGF and CTGF+ONA mice. Additionally, we performed RT-qPCR analyses with different microglia/macrophages and associated markers. Here, the mRNA levels of *Itgam*, which encodes for CD11b, *Cd68*, and *Nos2* were significantly upregulated in CTGF and CTGF+ONA retinae, while no alterations could be noticed for *Tgfb* mRNA levels, thereby confirming the prior results. Similar results were reported in a study by Sapienza et al., where IOP was elevated by cauterization of the episcleral veins. After 40 days, the loss of RGCs was accompanied by upregulated *Cd68* and *Nos2* mRNA levels (62). In general, reactive microglia/macrophages were found in a number of OHT (63–67) and normal-pressure animal models (68–70). The response of these cells often precedes neuronal cell loss. Especially in the EAG model, an increase in microglia/macrophage numbers was noted prior to RGC loss and optic nerve degeneration (68). Hence, we suggest that in our study presented here, the examined point in time was too late to detect any microglia/macrophage reaction in WT+ONA animals. On the other side, IOP elevation seems to trigger a more stable response of these cells. In human trabecular meshwork cells from primary open-angle glaucoma (POAG) patients, increased levels of genes associated with inflammation, including macrophages and T-cells, were found (71, 72). It is suggested that the disruption of the cytokine-mediated feedback loop in response to mechanical stress through elevated IOP is leading to chronic inflammation (73).

One of the cytokines produced by microglia/macrophages is TNF-α. Increased levels of this cytokine were found in the aqueous humor and serum of POAG patients (42, 43, 74, 75). TNF-α not only leads to a strong pro-inflammatory environment but can also be directly toxic to RGCs and axons (76–78). Our data revealed an upregulation of *Tnf* mRNA levels in CTGF and CTGF+ONA retina. Moreover, we conducted microarray analyses of serum samples. Here, no significant changes of TNF-α as well as its receptors TNF-receptor 1 + 2 were noted in all glaucoma mice. This might be due to the small sample size and should be repeated with more samples in the future. This could support prior research that suggests TNF-α serum levels as a possible disease marker for glaucoma patients (42, 43).

Both, TNF-α and macrophages can activate T-cells (79–81). Hence, we analyzed a possible T-cell response. By using flow cytometry, we were not able to detect a difference in the number of CD4^+^ cells in the retinae. It can be discussed that the cell population we detected by flow cytometry is triple positive for CD45, CD11b, and CD4 and hereby points towards a specific microglia population (82).

Factors such as inflammation, infection, or other pathological conditions, including glaucoma, can compromise the integrity of the blood-retinal barrier, leading to leakage. When the barrier is disrupted, various immune cells, including T-cells, may infiltrate the retina in response to the perceived damage. The activation of the complement cascade itself, as seen in our study, can lead to further vascular permeability through the generation of the anaphylatoxins C3a and C5a that facilitate blood-brain barrier breakdown in disorders of the central nervous system (83, 84). Intriguingly, by counting the CD3^+^ cells in retinal cross-sections, more T-cells were seen in the GCL of CTGF+ONA animals. Although the number of CD3^+^ cells was in general quite low, these results suggest a contribution of T-cells in the new multifactorial glaucoma model. The observed CD3^+^ cells do not seem to be co-localized with microglia/macrophages, although it is known that microglia interact with T-cells (85). In humans, the disbalance of Th1 and Th2 T-cells leads to a pro-inflammatory environment and in glaucoma donor eyes, CD3^+^ T-cells could be detected (44, 86, 87).

Recently, a study by Saini et al. revealed that Th1 cells specific for heat shock protein (HSP) 27, HSP60, and alpha-crystallin were significantly more abundant in peripheral blood monocytes from POAG patients compared to control samples. The higher counts of HSP-specific Th1 cells were associated with a thinner retinal nerve fiber layer in these patients (88). This research was based on a preceding animal study, where an HSP27-specific T-cell response was observed in an OHT mouse model (20). As mentioned, we observed only few CD3^+^ T-cells in the retinae and a higher number was only visible in the new CTGF+ONA model, while an increased response of microglia/macrophages and enhanced *Tnf* levels were also noted in CTGF and CTGF+ONA mice. While an immunization with ONA alone assumingly did not lead to a prolonged inflammation, the combination of autoimmune processes and high IOP triggered a T-cell response in these animals. It might seem contradictory that we detected few T-cells also in WT controls. However, the WT control animals in our study were also injected with pertussis toxin, which breaks down the blood-retina barrier. Similar results were previously noted in different animal models by other research groups as well as ours. These model include the EAG rat model (32), intravitreal injection of HSP27 (89), the experimental autoimmune encephalomyelitis model (90), or an OHT induction model (91).

The study presented here showed that immunological processes play a role in glaucomatous damage with and without IOP elevation. It is known that in the single models, namely EAG and CTGF, response of microglia/macrophages and an increase in complement system proteins could be observed prior to cell loss (8, 9, 68). Moreover, complement response was noted in a wave-like manner. Previously, we examined different points in time after ONA immunization, namely 3, 7, 14, and 28 days. While the number of C3^+^ and MAC^+^ cells were similar after 3 days, enhanced cell counts could be revealed at 7 days in ONA animals. 14 days post-immunization, the cell numbers went back to baseline level, while at 28 days, again more C3^+^ and MAC^+^ cells could be noted (8). This wave-like response is also likely in our current study. Conclusively, different points in time will be needed in the future to understand the immunological response in the novel CTGF+ONA model more precisely. Furthermore, it is noteworthy that we did not perfuse the animals before conducting the experiments. Therefore, some immune cells could have entered the retina from blood vessels. Further, for total cell quantification, counting beads could be added to the flow cytometry samples (92). This should be done in future studies, especially, when deciphering the role of T-cells in the CTGF+ONA mice in more detail.

Our results show that the immune response is not limited to the GCL but can also be observed in the IPL and INL. It is known that neuroinflammation can have direct damaging effects on RGCs and it also creates a pro-inflammatory environment and compromises the immune privilege of the retina (93). This, in turn, leads to the migration of immune cells into the retina. Moreover, resting microglia in the retina are most likely to be found in the plexiform layers (94). They migrate to the site of injury, e.g., towards RGCs, and further express e.g., complement proteins such as C1q. Moreover, amacrine cells can be affected by glaucoma. This could be shown in patients as well as in various animal models (31, 95–97). While mainly RGCs are damaged through glaucoma, also other cells in the retina can also be affected, especially, in later stages of the disease.

The combination of high-pressure CTGF mice and the normal-tension EAG model (CTGF+ONA) offers new opportunities to study the complex pathomechanisms of glaucoma disease and thus, will be helpful in finding novel therapeutic approaches. To even enhance this knowledge, a combination of the EAG model with other OHT or normal-tension models, including (magnetic) microbead (98, 99), silicone oil (100), laser photocoagulation (101), ischemic/reperfusion (102, 103), or optic nerve crush (104) should be performed prospectively.

## 5 Conclusion

In conclusion, this study underlines the contribution of the immune system in glaucoma disease and the interplay of complement system, microglia/macrophages, and T-cells. Moreover, we could highlight that an elevated IOP leads to a prolonged inflammation in the retina. In the new multifactorial glaucoma model, increased IOP and autoimmune processes seem to enforce an additional T-cell response. In future studies, examining different points in time could help to unravel the mechanisms in glaucoma disease more precisely and thus lead to better treatment options for patients prospectively.

## Data availability statement

The raw data supporting the conclusions of this article will be made available by the authors, without undue reservation.

## Ethics statement

The animal study was approved by Animal care committee of North Rhine-Westphalia. The study was conducted in accordance with the local legislation and institutional requirements.

## Author contributions

SR: Conceptualization, Formal analysis, Funding acquisition, Investigation, Project administration, Visualization, Writing – original draft. JW: Formal analysis, Funding acquisition, Investigation, Methodology, Visualization, Writing – review & editing. JT: Formal analysis, Investigation, Methodology, Visualization, Writing – review & editing. KS: Funding acquisition, Investigation, Methodology, Visualization, Writing – review & editing. MP: Formal analysis, Software, Supervision, Writing – review & editing. RF: Writing – review & editing. HD: Resources, Writing – review & editing. SJ: Project administration, Resources, Writing – review & editing.
